# Bioinformatics Analysis Reveals Cell Cycle-Related Gene Upregulation in Ascending Aortic Tissues From Murine Models

**DOI:** 10.3389/fgene.2022.823769

**Published:** 2022-03-08

**Authors:** Xiaoping Zhang, Zuozhen Yang, Xiaoyan Li, Xuxia Liu, Xipeng Wang, Tao Qiu, Yueli Wang, Tongxun Li, Qingle Li

**Affiliations:** ^1^ Beijing Anzhen Hospital, Capital Medical University, Beijing, China; ^2^ Beijing Institute of Heart, Lung and Blood Vessel Disease, Beijing, China; ^3^ MOE Laboratory of Biosystem Homeostasis and Protection, College of Life Sciences, Zhejiang University, Hangzhou, China; ^4^ Department of Vascular Surgery, Peking University People’s Hospital, Beijing, China; ^5^ The Second Affiliated Hospital of Guangzhou Medical University, Guangzhou, China

**Keywords:** Thoracic aortic aneurysm and dissection, bioinformatics analysis, ascending aortic tissues, cell cycle, inflammation

## Abstract

Thoracic aortic aneurysm and dissection (TAAD) is a high-risk aortic disease. Mouse models are usually used to explore the pathological progression of TAAD. In our studies, we performed bioinformatics analysis on a microarray dataset (GSE36778) and verified experiments to define the integrated hub genes of TAAD in three different mouse models. Gene Ontology (GO), Kyoto Encyclopedia of Genes and Genomes (KEGG) and protein–protein interaction (PPI) network analyses, and histological and quantitative reverse transcription-PCR (qRT–PCR) experiments were used in our study. First, differentially expressed genes (DEGs) were identified, and twelve common differentially expressed genes were found. Second, genes related to the cell cycle and inflammation were enriched by using GO and PPI. We focused on filtering and validating eighteen hub genes that were upregulated. Then, expression data from human ascending aortic tissues in the GSE153434 dataset were also used to verify our findings. These results indicated that cell cycle-related genes participate in the pathological mechanism of TAAD and provide new insight into the molecular mechanisms of TAAD.

## Introduction

Thoracic aortic aneurysm and dissection (TAAD) is a surgical emergency. When blood enters the medial layer through an intimal tear, rapid aortic rupture occurs (Bossone, et al., 2018). The onset of TAAD is usually sudden, without warning. Risk factors for TAAD include age, sex, hypertension, hypercholesterolemia, smoking and connective tissue disorders (Gawinecka, et al., 2017; Nienaber and Clough, 2015). In recent years, diagnostic and therapeutic techniques for TAAD have improved; however, its overall mortality remains high (Evangelista, et al., 2018; Melvinsdottir, et al., 2016). Therefore, research on the molecular mechanisms and pathological progression of TAAD is crucial. Many mouse models have been used in mechanistic studies, such as Fbn1 ^C1039G/+^ mice (Judge, et al., 2004) and mice with specific ablation of transforming growth factor-beta receptor II (Tgfbr2) in vascular smooth muscle cells (VSMC) (Li, et al., 2014).

**FIGURE 1:**
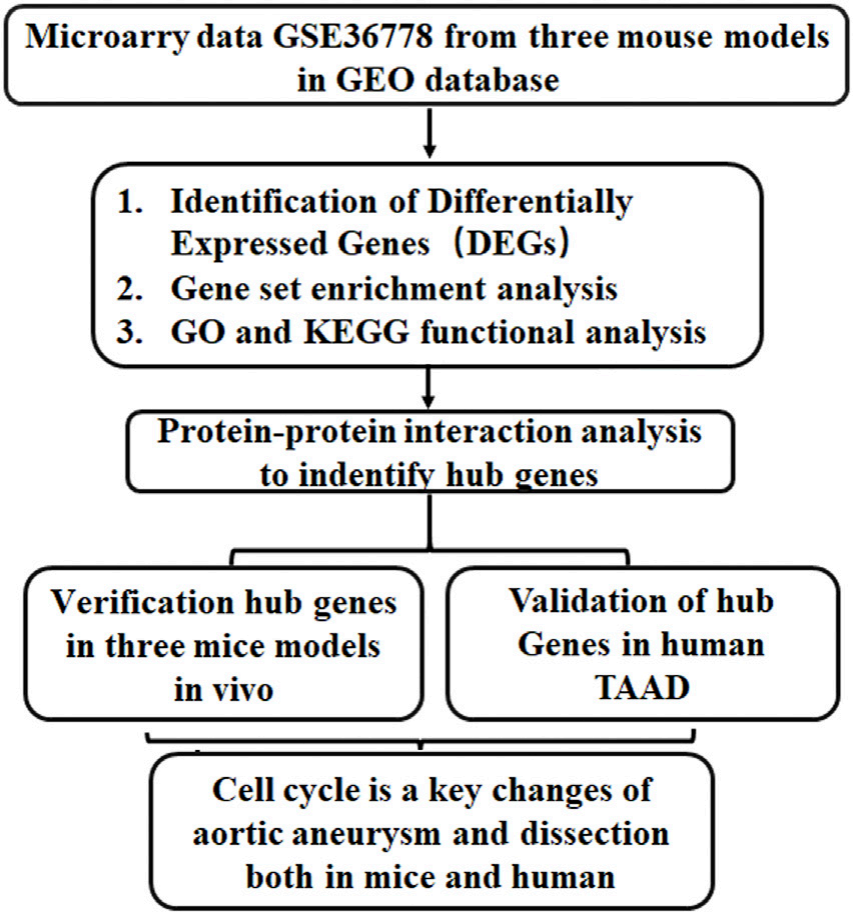
A schematic flowchart of the study. GO, Gene ontology; KEGG, Kyoto Encyclopedia of Genes and Genomes; TAAD, Thoracic aortic aneurysm and dissection.

Tgfbr2^f/f^ homozygous mice were generated through the insertion of loxP sites flanking TGFBR2 exon2 (Bhowmick, et al., 2004). Myh11-CreERT2 Tgfbr2^f/f^ mice were generated by crossing Myh11-cre (a smooth muscle-specific Myh11 promoter) mice and Tgfbr2 ^f/f^ mice, resulting in disease of the thoracic aorta (Li, et al., 2014). In mice with specific ablation of Tgfbr2 in the VSMCs at the age of 4 weeks, TAAD and hemorrhage of the aortic wall often occur in the ascending aorta and extend into arch branches. In previous studies, the loss of TGF-β signaling in VSMCs elicited vascular cell proliferation in all layers of the aortic wall (Li, et al., 2014), but the hub genes involved in the regulation of this process have not been revealed. Thus, to gain insights into hub gene regulation in the pathogenesis of TAAD progression, we performed bioinformatics analysis on a microarray dataset (GSE36778) and experimentally verified hub gene expression. A schematic flowchart of the techniques used is shown Figure 1.

In our research, bioinformatics analysis was applied to explore the gene expression profile of ascending aortic tissues from three murine models from GSE36778. Protein–protein interaction (PPI) analysis was performed to identify hub genes, which were enriched in cell cycle processes. Then, ascending aortic tissues from the same mouse models were collected, and the hub gene expression was validated using quantitative real-time reverse-transcription PCR (qRT–PCR). These studies highlighted and validated that cell cycle-related genes and immune cells were deregulated and explored the molecular mechanism involved in ascending aortic aneurysms.

## Materials and Methods

### Data Sourcing and Identification of Differentially Expressed Genes

The microarray dataset data (GSE36778) were downloaded from the GEO database (http://www.ncbi.nlm.nih.gov/geo/), basis on the GPL6246 platform analyzed by the Affymetrix Mouse Gene 1.0 ST Array. The datasets were 12 ascending aorta samples, including 3 Tgfbr2^f/f^ mice (CON), 3 Myh11-CreERT2. Tgfbr2^f/f^ mice (TR2), 3 Fbn1^C1039G/+^ mice (FB) and 3 Myh11-CreERT2. Tgfbr2^f/f^ mice/Fbn1^C1039G/+^ mice (FBTR2). FBTR2 mice is integrate specific ablation of TGF-β signaling in smooth muscle and heterozygous fibrillin-1 mutation.

Differentially expressed genes (DEGs) of three TAAD mice model comparison with CON was analyzed by GEO2R online tools (https://www.ncbi.nlm.nih.gov/geo/geo2r/), the R package (limma) was used for DGE identification, the data was transformed by log2 and the Benjamini & Hochberg (False discovery rate) was applied adjustment to the *p*-values. The change in threshold values were |log2 fold change (FC)| >0.58 and adjusted *p*-value<0.05 were considered significant for the DEGs. To show the differential expression of each DEG, volcano maps were drawn using the ggplot2 (R package) and heatmaps of the DEGs by pheatmap (R package).

### Gene Ontology and Kyoto Encyclopedia of Genes and Genomes Analyses

GO and KEGG enrichment analysis were analyzed by Metascape (https://metascape.org) for different changed genes. The picture of pathways was also generated by Metascape online tools. Ggplot2 (R package) was used to plot the KEGG pathways.

### Protein–Protein Interaction Network Detection

Top changed genes were selected for protein-protein interaction network by STRING database (https://www.string-db.org/) (Franceschini et al., 2012). The interaction network was visualized by in-house web-tool from STRING.

### Animal Models

Myh11-CreERT2. Tgfbr2^f/f^ mice and Fbn1^C1039G/+^ mice were obtained from *Jackson Lab*. All animals’ experiments were approved by the Institute of Institutional Animal Care and Use Committee of Capital Medical University, Beijing, China. Animal protocols were performed in accordance with the Guide for the Care and Use of Laboratory Animals published by the US National Institutes of Health. All the animals were housed in an environment with a temperature of 22 ± 1°C, relative humidity of 50 ± 1%, and a light/dark cycle of 12/12 h.

### Tamoxifen Injections

Tamoxifen solution was prepared by 40 mg tamoxifen (Sigma-Aldrich Corp, St. Louis, MO, United States; #T5648) was completely dissolved in 0.5 ml of 100% ethanol, and added 9.5 ml autoclaved olive oil, and stored at −20°C. Experimental mice at the age of 4 weeks injected 250 µl solution (1 mg/day tamoxifen) or of a control solution intraperitoneally into experimental mice each day for 5 consecutive days.

### Quantitative Reverse Transcription-PCR Validation

The hub genes were verified by Quantitative Reverse Transcription-PCR (qRT-PCR). Total RNA was reverse-transcribed to cDNA using First Strand cDNA Synthesis Kit (Life Technologies, Carlsbad, CA, United States) according to the manufacturer’s instructions. A CFX connected Real-time System (Bio-Rad, Hercules, CA, United States) and Green Premix ex Taq II (Takara, Dalian, China) was used for quantitative PCR. All primers used in this study are listed in Supplementary Table S1. All samples were normalized to GAPDH. And the relative expression levels of each gene were calculated using 2−ΔΔ^Ct^ methods.

### Histology

Aortas from CON, TR2, FB, and FBTR2 group mice were obtained for histopathological analysis. After mice were anesthetized, aortas were successively perfused by saline and 4% paraformaldehyde. Thoracic aortas were fixed for 24 h (4% paraformaldehyde), transferred to ethanol (70%) for subsequent dehydration and paraffin embedding. Aortas were continuously cut into sections, and staining Masson’s and EVG staining followed by protocol (Wang et al., 2021).

### Statistical Analysis

Data from 3 independent experiments were presented as means ± standard error of the mean (SEM). . Normally distributed data were compared by unpaired Student’s t-test for two groups comparisons and one-way analysis of variance (ANOVA). *p* values <0.05 were considered significant difference. Data statistically analysis were using GraphPad Prism 5.0 (GraphPad Software., San Diego, CA, United States) and SPSS software, version 23.0 (IBM Corp., Armonk, NY, United States).

## Results

### Identification of Differentially Expressed Genes

Twelve ascending aortic samples were subjected to comparative gene expression analysis among Tgfbr2^f/f^ mice (CON) and Myh11-CreERT2. Tgfbr2^f/f^ mice (TR2), Fbn1^C1039G/+^ mice (FB) and Fbn1^C1039G/+^/Myh11-CreERT2. Tgfbr2^f/f^ mice (FBTR2) at the age of 6 weeks (Li et al., 2014). TR2 and FBTR2 mice exhibited Tgfbr2-knockout VSMCs induced by tamoxifen at 4 weeks of age. All the normalized data are shown in box plots (data shown in Supplementary Figure S1).

**FIGURE 2:**
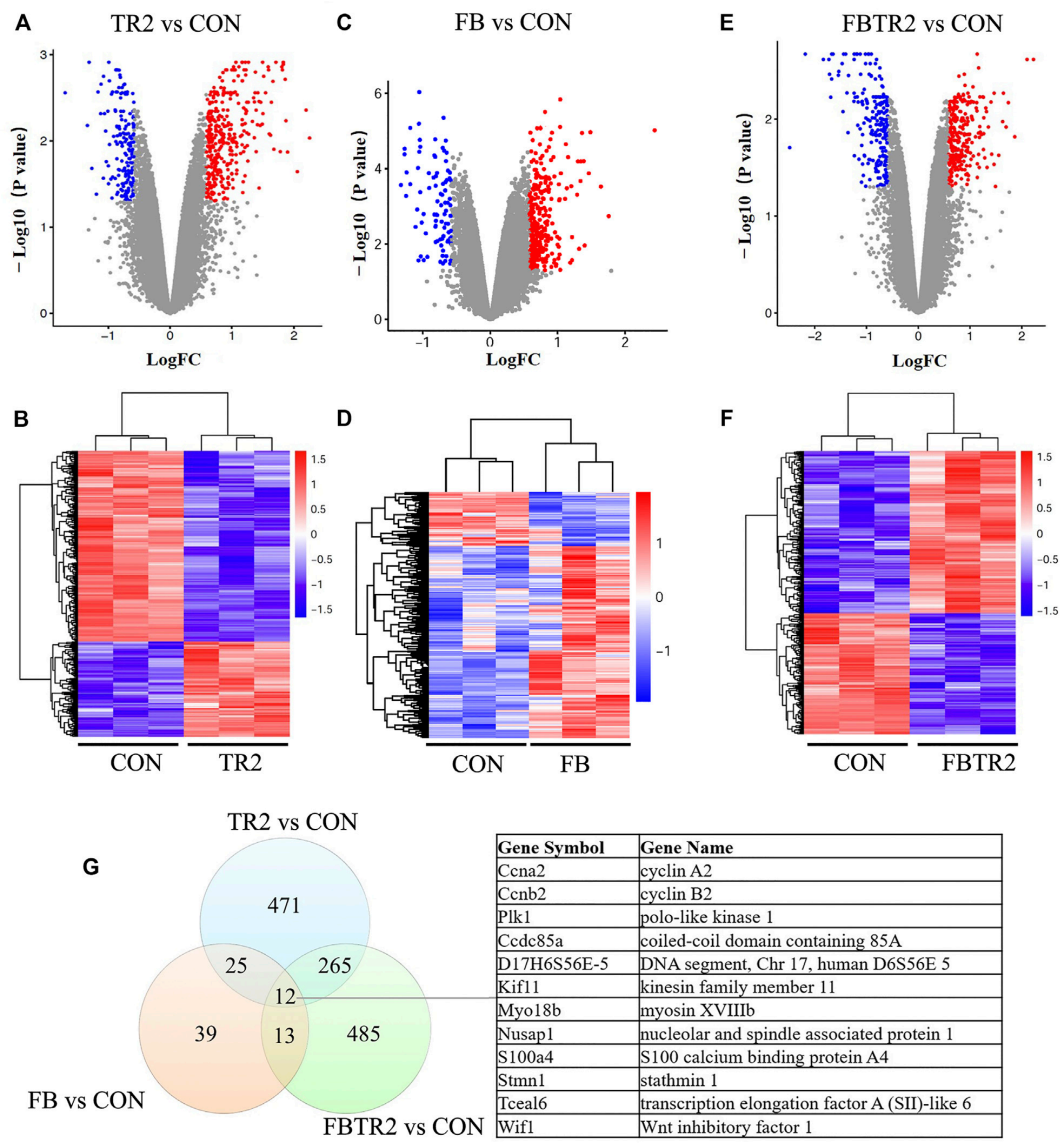
Identification of differentially expressed genes (DEGs). **(A)** Volcano plot and **(B)** Heatmap showing the expression of DEGs compared between TR2 and CON; red indicates up-regulated genes; blue indicates down-regulated genes. **(C)** Volcano plot and **(D)** Heatmap showing the expression of DEGs compared between FB and CON; **(E)** Volcano plot and **(F)** Heatmap showing the expression of DEGs compared between FBTR2 and CON; **(G)** Venn diagram showing the number of overlap genes in the three comparisons.

A total of 471 DEGs, including 349 upregulated genes and 122 downregulated genes, were identified by ascending aortic samples comparing the TR2 and CON mice (Figures 2A,B). A comparison of DEGs between these two groups showed that the number of significantly upregulated genes was higher than the number of significantly downregulated genes.

In contrast, 97 DEGs were obtained by comparing the FB and CON mice, including 60 upregulated genes and 37 downregulated genes (Figures 2C,D). Then, we identified 274 upregulated genes and 211 downregulated genes, for a total of 485 DEGs, in the comparison between FBTR2 and CON mice (Figures 2E,F).

We further analyzed the overlap DEGs between three mice models and CON mice. There were 25 DEGs common altered in both TR2 and FB mice, 265 mRNAs shift in TR2 and FBTR2 mice, 13 DEGs changed in FB and TR2FB mice. Moreover, 12 DEGs simultaneously varied in all three comparisons (8 upregulated and 4 downregulated genes) (Figure 2G). Three genes (Ccna2, Ccnb2, and Plk1) were involved in the cell cycle pathway, and the other 9 DEGs were Kif11, D17H6S56E-5, Ccdc85a, Myo18b, Nusap1, S100a4, Stmn1, Tceal6, and Wif1.

### Gene Ontology and Kyoto Encyclopedia of Genes and Genomes Functional Enrichment Analysis

**FIGURE 3:**
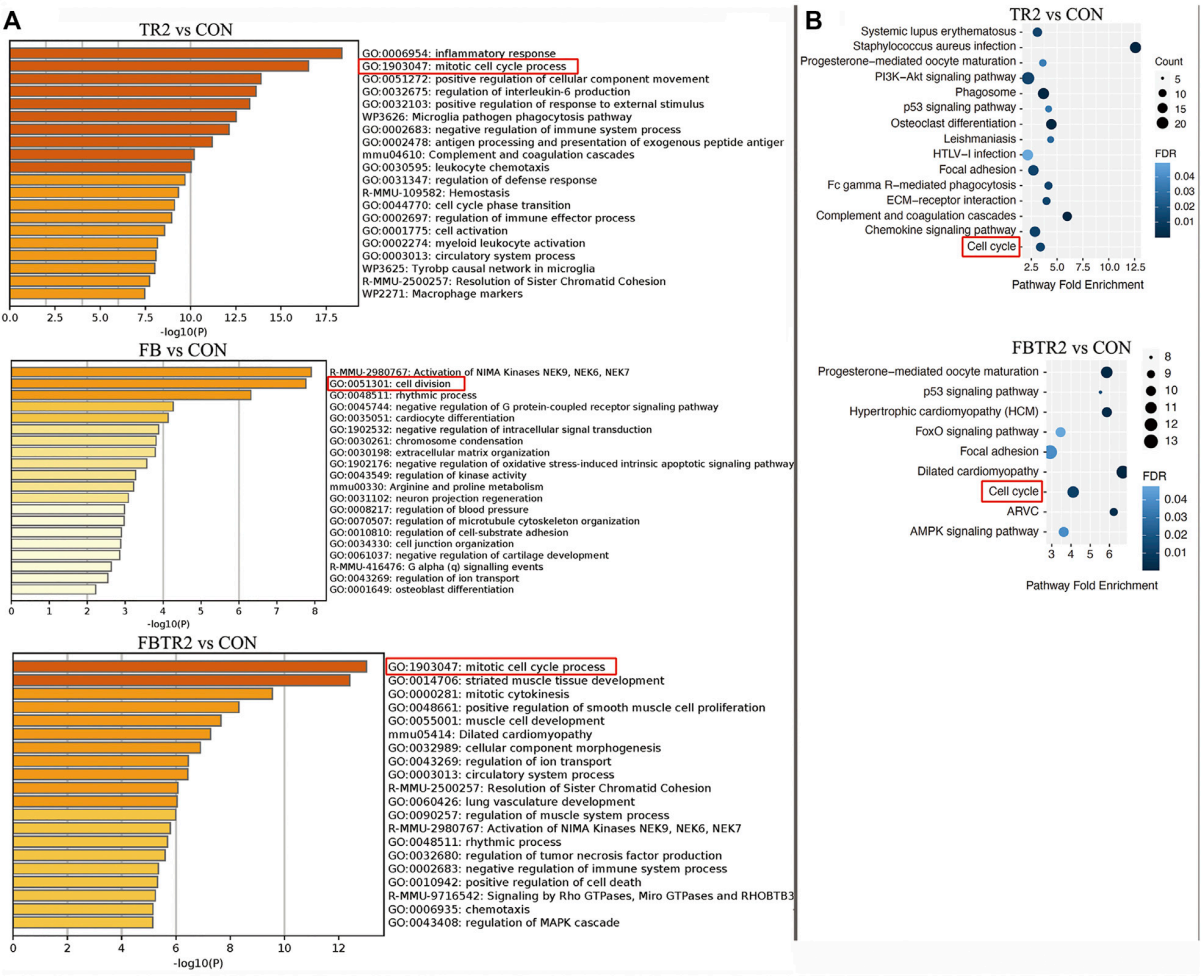
Gene ontology (GO) and Kyoto Encyclopedia of Genes and Genomes (KEGG) enrichment analysis. **(A)** GO enrichment analysis of DEGs in three comparison (TR2 vs. CON, FB vs. CON, FBTR2 vs. CON). **(B)** KEGG pathway enrichment analysis of DEGs compared between TR2, FBTR2 and CON.

To better understand the biological functions of mRNAs in ascending aortic samples from TAAD mice compared to wild-type mice, we performed GO and KEGG pathway enrichment analysis on DEGs. GO enrichment analysis was using Metascape online tools which is an effective and efficient tool for experimental biologists to comprehensively analyze in the big data era. During GO enrichment analysis, DEGs were mainly enriched for inflammatory response, mitotic cell cycle process, and regulation of interleukin-6 production in the comparison between TR2 and CON mice (Figure 3A). Furthermore, the comparison of FB and CON suggested that DEGs were enriched for Activation of NIMA Kinases, cell division, and rhythmic process (Figure 3A). Then, aberrantly expressed mRNAs between FBTR2 and CON were involved in mitotic cell cycle process, striated muscle tissue development, and mitotic cytokinesis (Figure 3A). Taken together, DEGs were enriched for mitotic cell cycle process and cell division in all three comparisons (Figure 3A). Cell cycle might be a key change in the ascending aortas of TAAD mice model.

KEGG pathway enrichment analysis demonstrated that the cell cycle and progesterone-mediated oocyte maturation pathways were the significant pathways shared among the three comparisons (Figure 3B).

### Protein–Protein Interaction Network Construction

**FIGURE 4:**
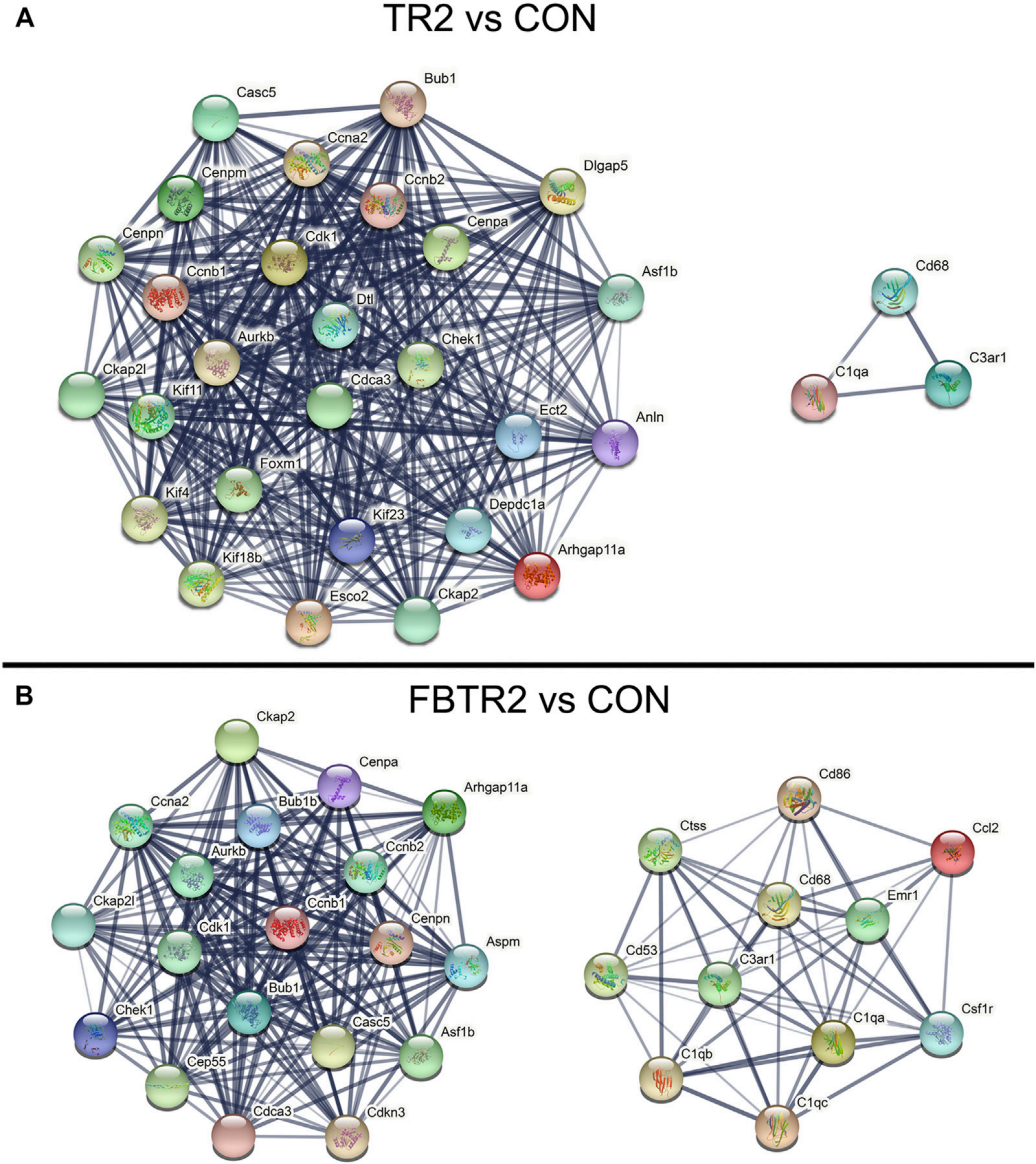
The protein–protein interaction (PPI) network constructed via the STRING database. **(A)** PPI network of the top 30 up-regulated DEGs compared between TR2 and CON, **(B)** PPI network of the top 30 up-regulated DEGs compared between FBTR2 and CON.

To explore the biological characteristics of these DEGs, PPI networks were constructed and analyzed by using the STRING online search tool (string-db.org, version info: 11.5) and compared between TR2 vs. CON (Figure 4A) and FBTR2 vs. CON (Figure 4B). The minimum required interaction score was selected as greater than 0.7. Two modules of the top 30 hub genes were filtered to show the functional and physical protein association network of all interacting factors. The significant nodes of the upregulated DEGs based on PPI network analysis contained 2 modules.

The PPI network in Figure 4A shows the comparison between TR2 vs. CON; the two modules are also shown in Figure 4B. The genes in the large module are related to the cell cycle, mitotic nuclear division, mitotic cytokinesis and cell division. The larger module included 27 genes, including Casc5, Cenpa, Cenpn, Cenpm, Ckap21, Kif11, Ccnb1, Ccnb2, Ccna2, Cdk1, Bub1, Aurkb, Dtl, Dlgap5, Cenpa, Asf1b, Chek1, Cdca3, Foxm1, Kif4, Kif18b, Kif23, Esco2, Ckap2, Depdc1a, Anln, and Arhgap11a. The small module included Cd68, C3ar1, and C1qa.

In the FBTR2 vs. CON comparison (Figure 4B), module 1 containing 19 genes was mainly relevant to the cell cycle and cell division and included Ckap2, Ccna2, Ckpa21, Chek1, Cep55, Cdk1, Aurkb, Bub1, Bub1b, Cenpa, Cenpn, Ccnb1, Ccnb2, Cdca3, Casc5, Aspm, Asf1b, Cdkn3, and Arhgap11a. Module 2 contained 11 genes mainly associated with immune system processes and the inflammatory response, including Ctss, Cd53, Cd86, Cd68, C3ar1, C1qb1, C1qc, C1qa, Emr1, Cccl2, and Csf1r. PPI network analysis was not performed between FB and CON comparisons because there were too few DEGs.

In the PPI network analysis, 15 integrated upregulated genes in two paired comparisons, Arhgap11a, Asf1b, Aurkb, Bub1, Casc5, Ccna2, Ccnb1, Ccnb2, Cdca3, Cdk1, Cenpa, Cenpn, Chek1, Ckap2, and Plk1, were identified as cell cycle-relevant modules. Three upregulated genes (C1qa, C3ar1, and Cd68) were inflammation genes. Then, GO and KEGG pathway enrichment analysis were performed for the 15 integrated upregulated genes and found that the most enriched pathways were the cell cycle, mitotic nuclear division and cell division (Table 1). Therefore, we identified 18 hub genes (Supplementary Table S2) that were relevant to the cell cycle and inflammation.

**TABLE 1:** GO functional annotation and KEGG pathway enrichment analysis of hub genes.

Category	Term Description	Genes	*p* Value
KEGG	Cell cycle	CCNA2, CCNB2, CCNB1, PLK1, CHEK1, CDK1, and BUB1	3.04E-11
	Progesterone-mediated oocyte maturation	CCNA2, CCNB2, CCNB1, PLK1, CDK1, and BUB1	1.71E-09
	Oocyte meiosis	CCNB2, CCNB1, PLK1, CDK1, and BUB1	9.40E-07
	P53 signaling pathway	CCNB2, CCNB1, CHEK1, and CDK1	1.73E-05
	FoxO signaling pathway	CCNB2, CCNB1, PLK1	0.005367
GO-BP	Mitotic nuclear division	CCNA2, CCNB2, CDCA3, PLK1, CDK1, CENPN, BUB1, and AURKB	2.24E-10
	Sister chromatid cohesion	PLK1, CENPN, CENPA, BUB1, and AURKB	9.15E-07
	G2/M transition of mitotic cell cycle	CCNB2, CCNB1, PLK1, CHEK1, and CDK1	2.86E-06
	Cell division	CCNA2, CCNB2, CCNB1, CDCA3, CDK1, and BUB1	4.29E-06
	Mitotic nuclear envelope disassembly	CCNB2, CCNB1, PLK1, and CDK1	4.71E-06
	Anaphase-promoting complex-dependent catabolic process	CCNB1, PLK1, CDK1, and AURKB	2.77E-05
	Regulation of ubiquitin-protein ligase activity involved in mitotic cell cycle	CCNB1, PLK1, and CDK1	1.39E-04
	Protein ubiquitination involved in ubiquitin-dependent protein catabolic process	CCNB1, PLK1, CDK1, and AURKB	1.98E-04
	Mitotic cytokinesis	PLK1, CKAP2, and CENPA	2.22E-04
	Cell proliferation	PLK1, CDK1, BUB1, and AURKB	0.002497

GO, Gene Ontology; KEGG, Kyoto Encyclopedia of Genes and Genomes; BP, biological process.

### Validation of Hub Genes by Quantitative Reverse Transcription-PCR

**FIGURE 5:**
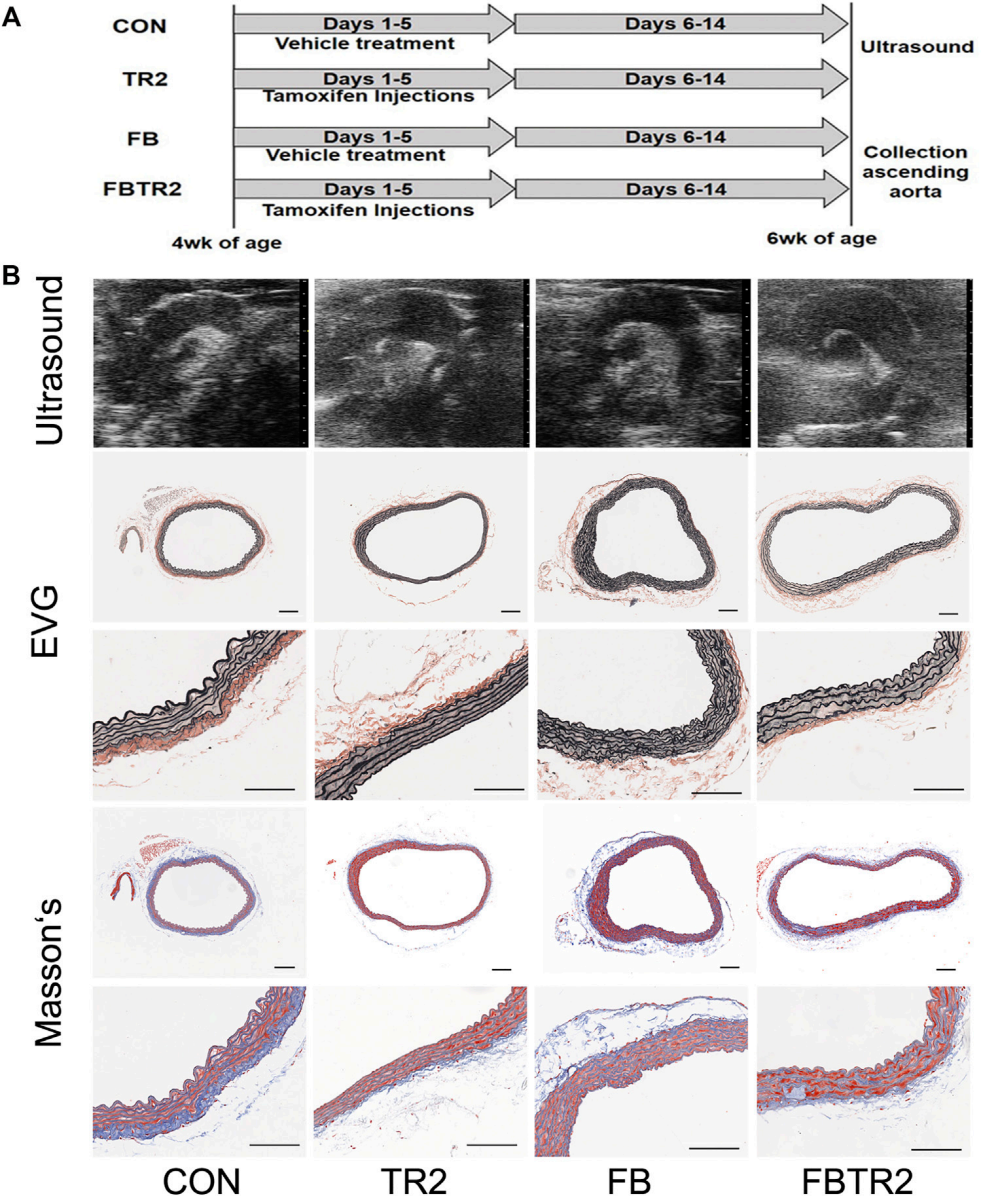
Representative images of ultrasound and histological results. **(A)** Experimental prcess of tamoxifen Injections and sample collection. **(B)** Representative images of ultrasound (upper), elastin Van Gieson staining (middle) and Masson’s staining (lower) results of Ascending Aortas from four group murine models. N = 6 mice per group. Scale bar, 50 μm.

For the validation of hub gene expression by qRT–PCR, ascending aortic samples from four groups of mice were collected in our lab (Figure 5A). HE and EVG staining were performed to investigate the histopathological changes in the ascending aortas of CON, TR2, FB and FBTR2 mice, as shown Figure 5B. Ultrasound investigation found that the ascending aortas were larger in TR2, FB, FBTR2 mice than in CON mice. Histological results showed that ascending aortic tissues significantly dilated aortic tissue in TR2, FB and FBTR2 mice (Figure 5B).

**FIGURE 6:**
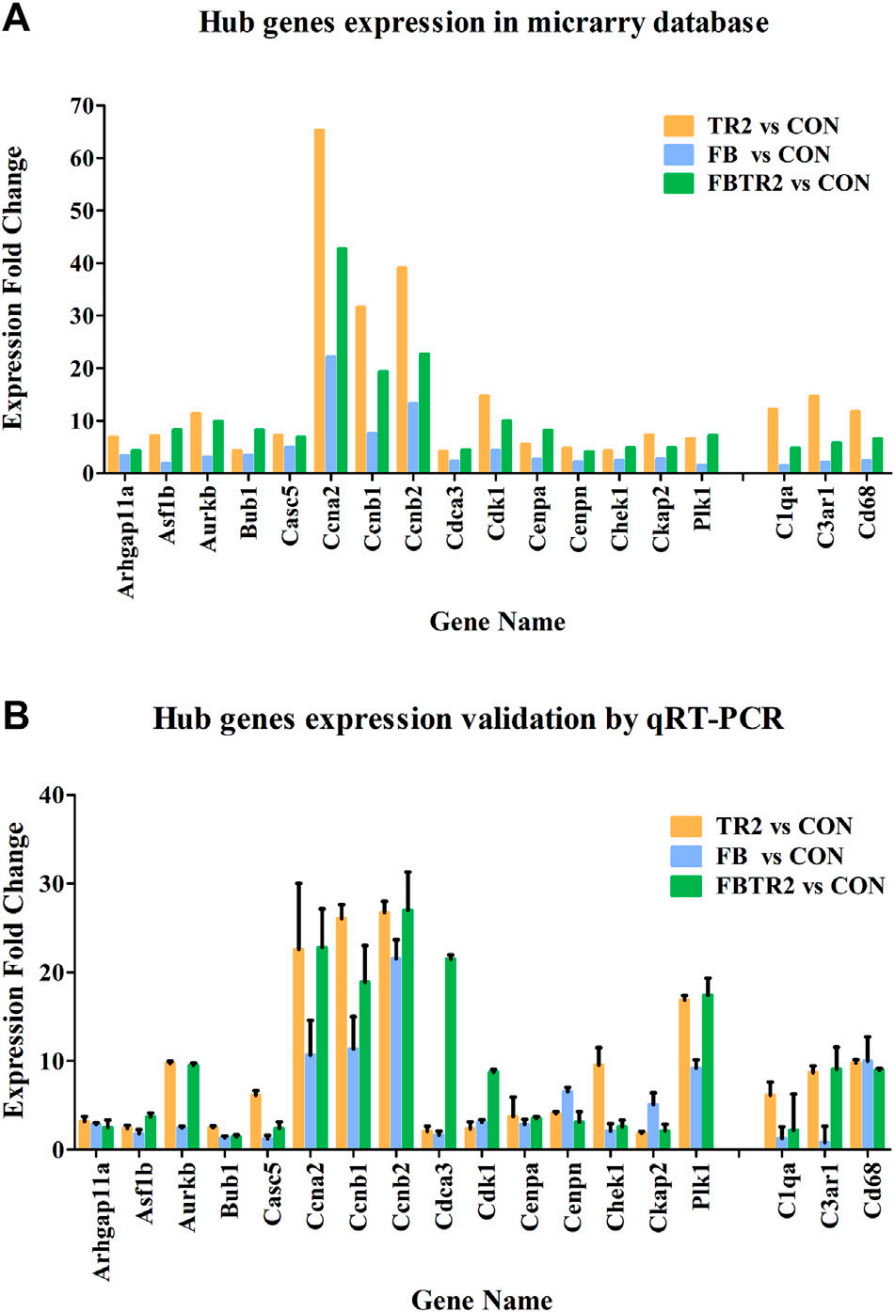
The Fold change of 18 Hub genes expression using microarray **(A)** and qRT-PCR **(B)**. Murine GAPDH was used as housekeeping internal control. Three independent experiments and duplicate every time were done in qRT-PCR.

To validate the microarray results, these 18 differentially expressed mRNAs were selected for qRT–PCR analysis in mouse aortae in the four groups (Figure 6B). All expression results detected by qRT–PCR were consistent with the microarray results (Figure 6A), indicating that the microarray data and analysis were highly reliable.

### Validation of Hub Genes in GSE153434

**FIGURE 7:**
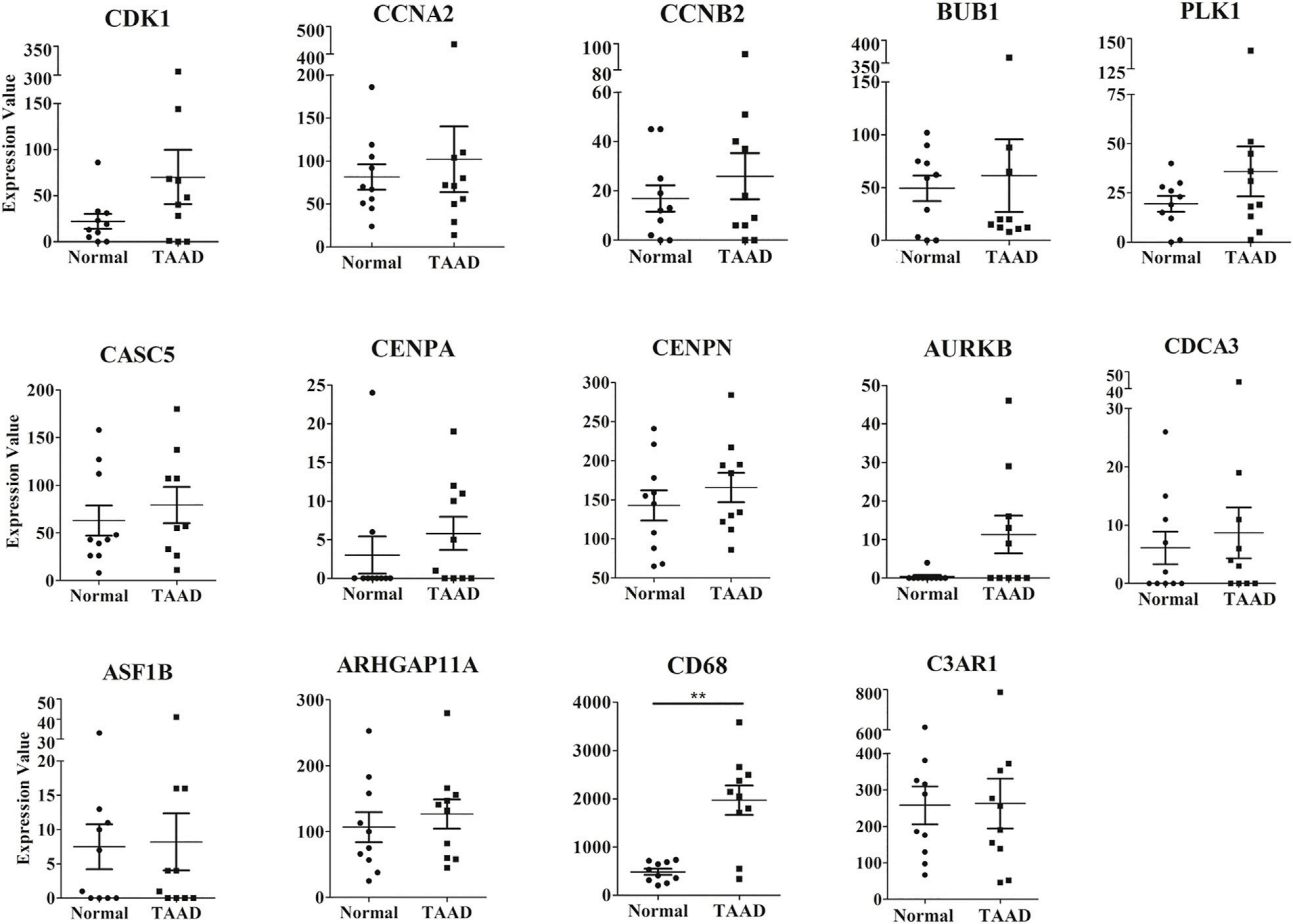
The expression levels of 14 hub genes between the TAAD and normal group in the GSE153434 datasets. **p < 0.01.

We validated the expression of 18 hub genes in GSE153434, which contained 10 TAAD aortic tissue samples and 10 normal aortic tissue samples (Zhou, et al., 2020). The expression of 14 of 18 hub genes was consistent with our analysis in mouse models: Arhgap11a, Asf1b, Aurkb, Bub1, Casc5, Ccna2, Ccnb2, Cdca3, Cdk1, Cenpn, Plk1, C3ar1, and Cd68 (Figure 7). Cd68 were significantly highly expressed in human TAAD samples. (Figure 7). The differences in these genes were not significant due to the small sample size and individual variability of the human aortic samples. These gene expression patterns were consistent with our results found in mice, indicating that hub genes may play roles in the pathogenesis of TAAD, not only in mice.

## Discussion

Much of the fundamental science of TAAD has focused on alterations in extracellular matrix components (Grewal and Gittenberger-de Groot, 2018), smooth muscle function (Rombouts, et al., 2021), and inflammation-related proteins (Gao, et al., 2021). According to research over the past several decades, TAAD development is the result of complex changes in the vascular cellular and extracellular environment and not a simple degenerative process (Del Porto, et al., 2013; He, et al., 2008; Malecki, et al., 2020; Sulkava, et al., 2017). To explore the molecular pathology of TAAD, many transcriptome sequences of TAAD samples from humans have been published, but these studies have had limitations (Bi, et al., 2020; Fang, et al., 2021; Gao, et al., 2021). Control aortic samples are difficult to obtain from healthy human specimens, and there is a high degree of individual variation among human specimens. Another limitation of studies of human TAAD tissue is that it is primarily carried out on end-stage surgical tissue, so the chronology of the inflammatory process is difficult to assess.

Our studies mined, sorted, and screened microarray data of three TAAD mouse models from the GSE36778 database using bioinformatics, and further histological and qRT–PCR methods were applied to validate the bioinformatics analysis results. Moreover, the hub genes identified in our studies were also validated in the human TAAD samples in the GSE153434 database.

To identify common differentially expressed genes, we compared the three TAAD mouse models with controls and identified 12 codifferentially expressed genes. Meanwhile, 3 of 12 integrated DEGs that were identified in three comparisons were cell cycle-related genes (Ccnb2, Ccna2, and Plk1). In view of the results of GO and KEGG term enrichment analysis, we found a strong relationship between the DEGs and the cell cycle, which was consistent with other studies (Wang, et al., 2019a).

In our study, the top 30 differentially expressed upregulated genes were filtered to reduce the scope of the study. The PPI network analysis showed that the 18 hub genes were divided into two modules: a larger module was associated with the cell cycle, and small modules were inflammation genes. Thus, GO, KEGG and PPI analyses all revealed that the cell cycle and inflammation were significantly upregulated, which provides full insights into aortic dissection.

Growing evidence suggests that inflammation participates in TAAD pathogenesis and the progression of disease (Gao, et al., 2021; He, et al., 2008). Notably, inflammation may occur early in the disease and is thus a driving factor (Goldfinger, et al., 2014; Wang, et al., 2021). Gene expression analysis revealed upregulation of multiple inflammatory pathways related to programmed cell death. Macrophages are present in human TAAD samples and play a critical role in the pathogenesis of TAAD (Del Porto, et al., 2013; Gao, et al., 2021). In our research, the inflammation-associated genes CD68, C1qa, and C3ar1 were hub genes. C1qa and C3ar1 are included in the complement system (Holt, et al., 2019), and CD68 is a marker of macrophages (He, et al., 2008). We validated these 3 gene changes by quantitative RT–PCR in TAAD mice compared with control mice. The complement system and inflammatory responses induced by macrophage infiltration enhance the pathological processes in TAAD.

Knowledge of the relationship between cell cycle-related proteins and TAAD pathological processes is limited. Upregulation of the Ccna2 and TP53 genes was observed in MFS aneurysm tissues (D’Amico, et al., 2020). Two previous bioinformatics studies (Wang, et al., 2019a; Wang, et al., 2019b) found that cell cycle-related genes (Cdk1, Ccnb1, Ccnb2, and Aurka) were upregulated in human TAAD samples and indicated that Cdk1 could be used as a potential diagnostic biomarker and therapeutic target of TAAD (Wang, et al., 2019a).

Previous studies also confirmed that a loss of TGF-β signaling in VSMCs elicits vascular cell proliferation in all layers of the aortic wall (Li, et al., 2014). Our results indicated that 15 cell cycle-related genes, Arhgap11a, Asf1b, Aurkb, Bub1, Casc5, Ccna2, Ccnb1, Ccnb2, Cdca3, Cdk1, Cenpa, Cenpn, Chek1, Ckap2, and Plk1, were all upregulated during TAAD development which are plays roles in vascular cell proliferation in aortic wall. Ccna2, Ccnb1, and Ccnb2 are members of the cyclin family and bind with cyclin-dependent kinase (Cdk1); the resulting complex drives cell cycle progression to promote cell proliferation (D'Amico, et al., 2020; de Carcer, et al., 2017). Asf1b (anti-silencing function 1b histone chaperone) plays roles in chromatin-based progression of cellular DNA replication and cell proliferation (Paul et al., 2016). Plk1 is an essential regulator of cell division (de Carcer, et al., 2017). A previous study found that Specific disruption of Plk1 in VSMCs led to impared arterial homeostasis in mice (Pal-Ghosh, et al., 2021). These genes included Plk1, Aurkb, Casc5, Cenpa, and Cenpn which are essential for spindle-assembly checkpoint signaling and for correct chromosome alignment (Frismantiene, et al., 2018). Cell-cycle relevant genes were essential for cell proliferation.

We identified the upregulation of cell cycle-relevant genes as a molecular characteristic of TAAD. A balance of vascular cell proliferation and death occurs in normal vessels (Liu, et al., 2021; Rayner, 2017). When vascular disease occurs, these carefully balanced pathways become disrupted (Rombouts, et al., 2021; Wang, et al., 2020). VSMCs in blood vessels retain a remarkable plasticity to maintain its biomechanical properties (Chakraborty, et al., 2021). VSMCs perform secretion and contractile roles in the health aortic vessels (Rzucidlo, et al., 2007). In response to vascular local environmental alterations in early stage of TAAD development, VSMCs changed differentiated/contractile phenotype, characterized by increased proliferation, migration, and extracellular matrix synthesis (Frismantiene, et al., 2018). These might indicate that cell cycle-relevant genes have a primary role in VSMC proliferation and phenotype changes, and promoting the progression of TAAD development.

We validation the expression patterns in human aortic samples, and found the expression of 14 of 18 hub genes was consistent with our analysis in mouse models but most not significance changes. The occurred differences were appeared might focus on two reasons. Firstly, Human tissues of TAAD which using for mRNA sequencing are one part of the intimal tear in patients with ascending aortas. However, ascending aortas in mice is small and using microarray detect entirely mRNA changes of ascending aortas. secondly, human ascending aortas cannot be excluded the factors that influence vascular remodeling from patient, such as age, sex, and weight. Different gene profiles of the same disease in human resulted in varying data. Mice TAAD models had better uniformity to help us declared the mechanism of TAAD disease. Bioinformatics analysis helped to obtain volumes of data in a short time and differential gene expression enrichment analysis would help obtain accurate direction and pave the way for future research. A limitation of this study was that the sample size of the GES36778 database was small, and the hub genes associated with the cell cycle need to be studied further to elucidate their roles in VSMC phenotype changes and other physiological processes.

## Conclusion

In conclusion, we identified differentially expressed genes in the ascending aorta common to three TAAD murine models that may be involved in the molecular pathology of TAAD. We also identified 18 hub genes by GO and PPI analysis. Cell cycle activation could be a molecular characteristic that provides new insights into the mechanism of TAAD. Further studies are needed to explore possible diagnostic biomarkers for TAAD.

## Data Availability

Publicly available datasets were analyzed in this study. This data can be found here: http://www.ncbi.nlm.nih.gov/geo/, GSE36778, GSE153434. All other data in the study are available on request from the corresponding author.

## Abbreviations

TAAD, Thoracic aortic aneurysm and dissection; GO, Gene Ontology; KEGG: Kyoto Encyclopedia of Genes and Genomes; qRT–PCR, quantitative reverse transcription–PCR; PPI: protein–protein interaction; VSMC, vascular smooth muscle cell; DEGs, differentially expressed genes.

## References

[B1] Bhowmick N. A. Chytil A. Plieth D. Gorska A. E. Dumont N. Shappell S. (2004). TGF-ß Signaling in Fibroblasts Modulates the Oncogenic Potential of Adjacent Epithelia. Science 303 (5659), 848–851. 10.1126/science.1090922 14764882

[B2] Bi S. Liu R. Shen Y. Gu J. (2020). Bioinformatics Analysis of Key Genes and miRNAs Associated with Stanford Type A Aortic Dissection. J. Thorac. Dis. 12 (9), 4842–4853. 10.21037/jtd-20-1337 33145057 PMC7578500

[B3] Bossone E. LaBounty T. M. Eagle K. A. (2018). Acute Aortic Syndromes: Diagnosis and Management, an Update. Eur. Heart J. 39 (9), 739–749d. 10.1093/eurheartj/ehx319 29106452

[B4] Chakraborty A. Li Y. Zhang C. Li Y. LeMaire S. A. Shen Y. H. (2022). Programmed Cell Death in Aortic Aneurysm and Dissection: A Potential Therapeutic Target. J. Mol. Cel Cardiol 163, 67–80. 10.1016/j.yjmcc.2021.09.010 PMC8816882 34597613

[B5] D’Amico F. Doldo E. Pisano C. Scioli M. G. Centofanti F. Proietti G. (2020). Specific miRNA and Gene Deregulation Characterize the Increased Angiogenic Remodeling of Thoracic Aneurysmatic Aortopathy in Marfan Syndrome. Ijms 21, 6886. 10.3390/ijms21186886 PMC7555983 32961817

[B6] de Cárcer G. Wachowicz P. Martínez-Martínez S. Oller J. Méndez-Barbero N. Escobar B. (2017). Plk1 Regulates Contraction of Postmitotic Smooth Muscle Cells and Is Required for Vascular Homeostasis. Nat. Med. 23 (8), 964–974. 10.1038/nm.4364 28692064

[B7] Del Porto F. di Gioia C. Tritapepe L. Ferri L. Leopizzi M. Nofroni I. (2013). The Multitasking Role of Macrophages in Stanford Type A Acute Aortic Dissection. Cardiology 127 (2), 123–129. 10.1159/000355253 24334970

[B8] Evangelista A. Isselbacher E. M. Bossone E. Gleason T. G. Eusanio M. D. Sechtem U. (2018). Insights from the International Registry of Acute Aortic Dissection. Circulation 137 (17), 1846–1860. 10.1161/circulationaha.117.031264 29685932

[B9] Fang J. Pan Z. Yu H. Yang S. Hu X. Lu X. (2021). Regulatory Master Genes Identification and Drug Repositioning by Integrative mRNA-miRNA Network Analysis for Acute Type A Aortic Dissection. Front. Pharmacol. 11, 575765. 10.3389/fphar.2020.575765 33551796 PMC7861055

[B10] Franceschini A. Szklarczyk D. Frankild S. Kuhn M. Simonovic M. Roth A. (2012). STRING v9.1: Protein-Protein Interaction Networks, with Increased Coverage and Integration. Nucleic Acids Res. 41 (Database issue), D808–D815. 10.1093/nar/gks1094 23203871 PMC3531103

[B11] Frismantiene A. Philippova M. Erne P. Resink T. J. (2018). Smooth Muscle Cell-Driven Vascular Diseases and Molecular Mechanisms of VSMC Plasticity. Cell Signal. 52, 48–64. 10.1016/j.cellsig.2018.08.019 30172025

[B12] Gao H. Sun X. Liu Y. Liang S. Zhang B. Wang L. (2021). Analysis of Hub Genes and the Mechanism of Immune Infiltration in Stanford Type a Aortic Dissection. Front. Cardiovasc. Med. 8, 680065. 10.3389/fcvm.2021.680065 34277731 PMC8284479

[B13] Gawinecka J. Schonrath F. von Eckardstein A. (2017). Acute Aortic Dissection: Pathogenesis, Risk Factors and Diagnosis. Swiss Med. Wkly 147, w14489. 10.4414/smw.2017.14489 28871571

[B14] Goldfinger J. Z. Halperin J. L. Marin M. L. Stewart A. S. Eagle K. A. Fuster V. (2014). Thoracic Aortic Aneurysm and Dissection. J. Am. Coll. Cardiol. 64 (16), 1725–1739. 10.1016/j.jacc.2014.08.025 25323262

[B15] Grewal N. Gittenberger-de Groot A. C. (2018). Pathogenesis of Aortic wall Complications in Marfan Syndrome. Cardiovasc. Pathol. 33, 62–69. 10.1016/j.carpath.2018.01.005 29433109

[B16] He R. Guo D.-C. Sun W. Papke C. L. Duraisamy S. Estrera A. L. (2008). Characterization of the Inflammatory Cells in Ascending Thoracic Aortic Aneurysms in Patients with Marfan Syndrome, Familial Thoracic Aortic Aneurysms, and Sporadic Aneurysms. J. Thorac. Cardiovasc. Surg. 136 (4), 922–929. 10.1016/j.jtcvs.2007.12.063 18954631 PMC2590650

[B17] Holt M. Seim B. E. Øgaard J. Olsen M. B. Woldbæk P. R. Kvitting J. P. (2019). Selective and Marked Decrease of Complement Receptor C5aR2 in Human Thoracic Aortic Aneurysms: a Dysregulation with Potential Inflammatory Effects. Open Heart 6, e001098. 10.1136/openhrt-2019-001098 31798913 PMC6861114

[B18] Judge D. P. Biery N. J. Keene D. R. Geubtner J. Myers L. Huso D. L. (2004). Evidence for a Critical Contribution of Haploinsufficiency in the Complex Pathogenesis of Marfan Syndrome. J. Clin. Invest. 114 (2), 172–181. 10.1172/jci200420641 15254584 PMC449744

[B19] Li W. Li Q. Jiao Y. Qin L. Ali R. Zhou J. (2014). Tgfbr2 Disruption in Postnatal Smooth Muscle Impairs Aortic wall Homeostasis. J. Clin. Invest. 124 (2), 755–767. 10.1172/jci69942 24401272 PMC3904608

[B20] Liu C. X. Tan Y. Z. Deng C. Q. (2021). Main Active Components and Cell Cycle Regulation Mechanism of Astragali Radix and Angelicae Sinensis Radix in the Treatment of Ox-LDL-Induced HUVECs Injury and Inhibition of Their Cell Cycle. Evid. Based Complement. Alternat Med. 2021, 8087183. 10.1155/2021/8087183 34471419 PMC8405292

[B21] Malecki C. Hambly B. D. Jeremy R. W. Robertson E. N. (2020). The Role of Inflammation and Myeloperoxidase-Related Oxidative Stress in the Pathogenesis of Genetically Triggered Thoracic Aortic Aneurysms. Int. J. Mol. Sci. 21 (20), 7678. 10.3390/ijms21207678 PMC7590002 33081376

[B22] Melvinsdottir I. H. Lund S. H. Agnarsson B. A. Sigvaldason K. Gudbjartsson T. Geirsson A. (2016). The Incidence and Mortality of Acute Thoracic Aortic Dissection: Results from a Whole Nation Study. Eur. J. Cardiothorac. Surg. 50 (6), 1111–1117. 10.1093/ejcts/ezw235 27334108

[B23] Nienaber C. A. Clough R. E. (2015). Management of Acute Aortic Dissection. The Lancet 385 (9970), 800–811. 10.1016/s0140-6736(14)61005-9 25662791

[B24] Pal-Ghosh R. Xue D. Warburton R. Hill N. Polgar P. Wilson J. L. (2021). CDC2 Is an Important Driver of Vascular Smooth Muscle Cell Proliferation via FOXM1 and PLK1 in Pulmonary Arterial Hypertension. Int. J. Mol. Sci. 22 (13), 6943. 10.3390/ijms22136943 34203295 PMC8268698

[B25] Paul P. K. Rabaglia M. E. Wang C.-Y. Wang C.-Y. Stapleton D. S. Leng N. (2016). Histone Chaperone ASF1B Promotes Human β-cell Proliferation via Recruitment of Histone H3.3. Cell Cycle 15 (23), 3191–3202. 10.1080/15384101.2016.1241914 27753532 PMC5176155

[B26] Rayner K. J. (2017). Cell Death in the Vessel Wall: The Good, the Bad, the Ugly. Arterioscler Thromb. Vasc. Biol. 37 (7), e75–e81. 10.1161/ATVBAHA.117.309229 28637702 PMC5584709

[B27] Rombouts K. B. van Merrienboer T. A. R. Ket J. C. F. Bogunovic N. van der Velden J. Yeung K. K. (2021). The Role of Vascular Smooth Muscle Cells in the Development of Aortic Aneurysms and Dissections. Eur. J. Clin. Invest., e13697. 10.1111/eci.13697 34698377 PMC9285394

[B28] Rzucidlo E. M. Martin K. A. Powell R. J. (2007). Regulation of Vascular Smooth Muscle Cell Differentiation. J. Vasc. Surg. Suppl. A. 45 Suppl A, A25–A32. 10.1016/j.jvs.2007.03.001 17544021

[B29] Sulkava M. Raitoharju E. Mennander A. Levula M. Seppälä I. Lyytikäinen L. P. (2017). Differentially Expressed Genes and Canonical Pathways in the Ascending Thoracic Aortic Aneurysm - the Tampere Vascular Study. Sci. Rep. 7 (1), 12127. 10.1038/s41598-017-12421-4 28935963 PMC5608723

[B30] Wang W. Liu Q. Wang Y. Piao H. Li B. Zhu Z. (2019a). Verification of Hub Genes in the Expression Profile of Aortic Dissection. PLoS One 14 (11), e0224922. 10.1371/journal.pone.0224922 31751374 PMC6872142

[B31] Wang W. Wang T. Wang Y. Piao H. Li B. Zhu Z. (2019b). Integration of Gene Expression Profile Data to Verify Hub Genes of Patients with Stanford A Aortic Dissection. Biomed. Res. Int. 2019, 3629751. 10.1155/2019/3629751 31380418 PMC6662449

[B32] Wang X. Zhang X. Qiu T. Yang Y. Li Q. (2021). Dexamethasone Reduces the Formation of Thoracic Aortic Aneurysm and Dissection in a Murine Model. Exp. Cel Res. 405 (2), 112703. 10.1016/j.yexcr.2021.112703 34118251

[B33] Wang Z. Qi Y. Wang R. Wu W. Li Z. Wang M. (2020). IGFBP6 Regulates Vascular Smooth Muscle Cell Proliferation and Morphology via Cyclin E-CDK2. J. Cel Physiol 235 (12), 9538–9556. 10.1002/jcp.29762 32529639

[B34] Zhou Z. Liu Y. Zhu X. Tang X. Wang Y. Wang J. (2020). Exaggerated Autophagy in Stanford Type A Aortic Dissection: A Transcriptome Pilot Analysis of Human Ascending Aortic Tissues. Genes (Basel) 11 (10). 10.3390/genes11101187 PMC7650806 33066131

