# Supplementary material for: Bioinformatics Analysis Reveals Cell Cycle-Related Gene Upregulation in Ascending Aortic Tissues From Murine Models
Source: Front Genet. 2022 Mar 8;13:823769. doi: 10.3389/fgene.2022.823769 (PMC8959095; doi:10.3389/fgene.2022.823769)
Supplement: Supplementary file 1 [file DataSheet1.PDF]

Table S1. The Mouse Primer Sequences Used for qRT-PCR.

Gene Name	Forward primer (5′–3′)	Reverse primer (5′–3′)
Arhgap11a	GTCTATGGCATTAAGGTCAAGGG	TCTGGAACGACTGAATGAGGC
Asf1b	CCTTCCGGTTCGAGATCAGC	GGATGGGTTTGGGGCATCAG
Aurkb	CAGAAGGAGAACGCCTACCC	GAGAGCAAGCGCAGATGTC
Bub1	AGAATGCTCTGTCAGCTCATCT	TGTCTTCACTAACCCACTGCT
Casc5	AGAGACACAGAAAACGCAGAC	ACATCCCACTCAGACAAACTCA
Ccna2	GCCTTCACCATTCATGTGGAT	TTGCTGCGGGTAAAGAGACAG
Ccnb1	AAGGTGCCTGTGTGTGAACC	GTCAGCCCCATCATCTGCG
Ccnb2	GCCAAGAGCCATGTGACTATC	CAGAGCTGGTACTTTGGTGTTC
Cdca3	GAGTAGCAGACCCTCGTTCAC	TCTCTACCTGAATAGGAGTGCG
Cdk1	AGAAGGTACTTACGGTGTGGT	GAGAGATTTCCCGAATTGCAGT
Cenpa	CTCCAGTGTAGGCTCTCAGAC	CTGAAAGGCTTCTTCCTGAACA
Cenpn	TGAGGGTCCACCGAGTCAC	CCCCCACCTATGTTACTCTTGA
Chek1	GTTAAGCCACGAGAATGTAGTGA	GATACTGGATATGGCCTTCCCT
Ckap2	ACATCTGAGGATCGGGTCCAA	ACCTGGCAAATCATCTCTTGTAG
Plk1	CTTCGCCAAATGCTTCGAGAT	CTTCGCCAAATGCTTCGAGAT
C1qa	AAAGGCAATCCAGGCAATATCA	TGGTTCTGGTATGGACTCTCC
C3ar1	TCGATGCTGACACCAATTCAA	TCCCAATAGACAAGTGAGACCAA
Cd68	TGTCTGATCTTGCTAGGACCG	GAGAGTAACGGCCTTTTTGTGA

Figure. S1

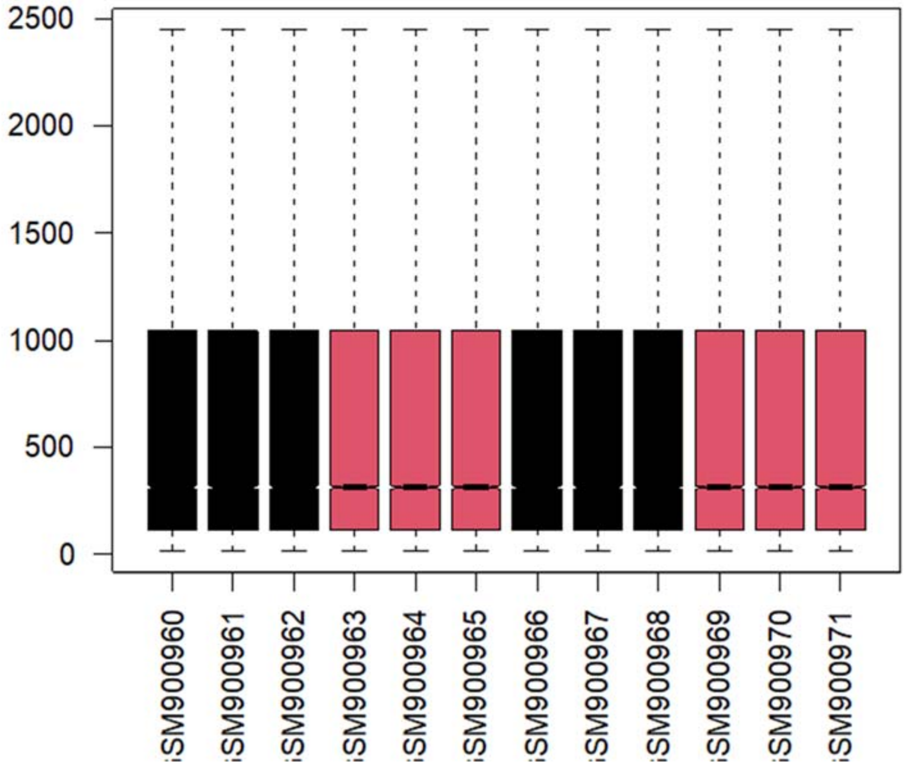

Figure.S1. Data processing of gene expression profiles. Microarray data from GES was normalization and background correction. The represent data from CON, TR2, FB and FBTR2 mice.

TABLE S2. Hub Gene Description.

Gene symbol	Description	Category	Ensembl ID
Arhgap11a	Rho GTPase Activating Protein 11A	Protein coding	ENSG00000198826
Asf1b	Anti-Silencing Function 1B Histone Chaperone	Protein coding	ENSG00000105011
Aurkb	Aurora Kinase B	Protein coding	ENSG00000178999
Bub1	BUB1 Mitotic Checkpoint Serine/Threonine Kinase	Protein coding	ENSG00000169679
Casc5	cancer susceptibility candidate 5, also known as Knl1, kinetochore scaffold 1	Protein coding	ENSG00000137812
Ccna2	Cyclin A2	Protein coding	ENSG00000145386
Ccnb1	Cyclin B1	Protein coding	ENSG00000134057
Ccnb2	Cyclin B2	Protein coding	ENSG00000157456
Cdca3	Cell Division Cycle Associated 3	Protein coding	ENSG00000111665
Cdk1	Cyclin Dependent Kinase 1	Protein coding	ENSG00000170312
Cenpa	Centromere Protein A	Protein coding	ENSG00000115163
Cenpn	Centromere Protein A	Protein coding	ENSG00000166451
Chek1	Checkpoint Kinase 1	Protein coding	ENSG00000149554
Ckap2	Cytoskeleton Associated Protein 2	Protein coding	ENSG00000136108
Plk1	Polo Like Kinase 1	Protein coding	ENSG00000166851
CD68	CD68 Molecule	Protein coding	ENSG00000129226
C1qa	Complement C1q A Chain	Protein coding	ENSG00000173372
C3ar1	complement C3a receptor 1	Protein coding	ENSG00000171860
